# The Potential of West Nile Virus Transmission Regarding the Environmental Factors Using Geographic Information System (GIS), West Azerbaijan Province, Iran

**Published:** 2019-03-30

**Authors:** Mojtaba Amini, Ahmad Ali Hanafi-Bojd, Sayyad Asghari, Ali Reza Chavshin

**Affiliations:** 1 Department of Medical Entomology and Vector Control, School of Public Health, Urmia University of Medical Sciences, Urmia, Iran; 2 Department of Medical Entomology and Vector Control, School of Public Health, Tehran University of Medical Sciences, Tehran, Iran; 3 Department of Geography, School of Humanities, University of Mohaghegh Ardabili, Ardabil, Iran; 4 Social Determinants of Health Research Center, Urmia University of Medical Sciences, Urmia, Iran

**Keywords:** Mosquitoes, *Aedes caspius*, *Culex pipiens*, Iran

## Abstract

**Background:**

West Nile fever, as an expanding zoonotic disease, has been reported from different creatures involved in the disease from Iran. In addition to biological mosquito-associated factors, various elements such as their activities, distribution, behavior and vectorial capacity could be affected by environmental factors. We determined the distribution of West Nile virus (WNV) vectors, the environmental factors affecting WNV transmission and the high-risk areas across West Azerbaijan Province (Northwestern Iran), regarding the potential of WNV transmission using Geographical Information System (GIS).

**Methods:**

Mosquitoes’ larvae and adults were collected from different habitats of the province in 2015 and identified using standard morphological keys. The data regarding the distribution of mosquitoes across the studied area were organized in ArcMap databases. Inverse Distance Weighted (IDW) interpolation analysis was conducted on the data of synoptic stations to find climatic variables in the collection sites of different mosquito species. Layers of transmission-related environmental factors were categorized and weighed based on their effects on disease transmission.

**Results:**

Overall, 2813 samples of different mosquito species from different regions of the province were collected and identified. According to the GIS analysis, areas in the northeastern province, which have lower altitudes and slopes with higher temperatures and more water bodies, were found to have better condition for the activity of mosquitoes (as high-risk areas: hot spots).

**Conclusion:**

The precision of our results was proven to be in line with previous study results that identified high-risk areas, where WNV-infected vectors were captured from these same areas.

## Introduction

Due to notable problems caused by mosquitoes and mosquito-borne diseases (MBDs), the studies of factors influencing the presence, activities, and distribution of mosquitoes and MBDs are important and form absolute parts of the epidemiology of MBDs, in which environmental conditions and their changes are components of this process (1). In addition to biological factors, mosquitoes’ activities, distribution, behavior, and even their vectorial capacity could be affected by environmental conditions (2–5).

Mosquitoes’ feeding rates vary expressively with temperature. Moreover, feeding behavior and host availability are affected by climate. Additionally, length of the gonotrophic cycle as an important factor in diseases transmission could be influenced by precipitation patterns (6). Finally, a positive correlation between temperature and West Nile and West Nile virus (WNV) incidence in mosquitoes have been reported (7, 8).

Among the different tools used to identify the effects of environmental factors on MBDs and their epidemiology, is Geographical Information System (GIS) (9). In addition, global positioning systems (GPS), remote sensing, and spatial statistics could also play important role in MBDs research, surveillance, and control programs (10).

Among the MBDs, West Nile fever is an emerging zoonosis rapidly spread and caused expansive health threat in different parts of the world (11, 12). The transmission cycle of WNV includes a wide range of migratory birds as reservoirs (13), equines (14) and humans (15) as dead-end hosts, and numerous mosquito species including different species e.g. *Culex pipiens*, *Cx. restuans*, *Cx. salinarius*, *Cx. tarsalis*, *Aedes vexans*, *Ae. albopictus*, and *Coquillettidia perturbans* as biological vectors (16–18). WNV has been isolated and identified in numerous mosquito species including different species of *Culex*, *Aedes*, and *Anopheles* (19–21). Due to the wide use of GIS in the study of vector-borne diseases (including MBDs) (22, 23), several studies have employed GIS across the world to predict, risk assessment (18), surveillance (25, 26) and the environmental factors influencing WNV transmission (27, 28).

Because of the presence of theoretically favorable environments for the establishment of WNV across Iran, the presence of WNV has been investigated and showed the sero-prevalence rate of (1.3%) in human (29–33), 23.7% in equines (34), overall (15%) infection in birds (35) and recently among the potential vector species, in which among them WNV has been isolated and reported from *Ae. caspius* (36) and *Culex* spp (37).

Iranian West Azerbaijan Province in the northwestern part of the country, could be considered as one of the most suspicious areas in Iran regarding the possibility of establishment and transmission of WNV, because of its abundant water resources and wetlands for migratory birds from different parts around the world, which serve as reservoirs of WNV (38, 39) and also, the presence of potential vector species of mosquitoes in this region e.g. *Culex pipiens* s.l., *Ae. caspius*, *Anopheles maculipennis* s.l., *Culiseta longiareolata* (40–42). As the first isolation of WNV from its potential vectors was reported from this region (36), and this region borders several countries such as Turkey, Iraq, Armenia, and the Republic of Azerbaijan, more attention is needed at this area.

Due to the special circumstances mentioned above about West Azerbaijan Province, we aimed to determine: 1) the distribution of probable WNV vectors, 2) the environmental factors affecting WNV transmission and 3) the high-risk areas across the province regarding the potential of WNV transmission using GIS.

## Materials and Methods

### Study area

**Fig. 1.**
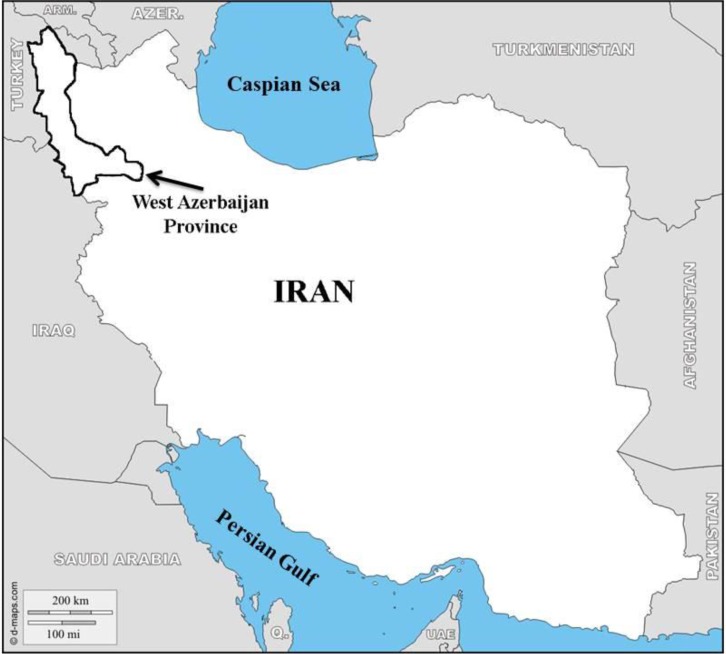
Study area in northwestern Iran

West Azerbaijan Province is located in the northwest of Iran between latitudes 35° 58′–39° 46′ N and longitudes 44° 3′–47° 23′ E. This province formally includes 17 counties. It is bordered by Turkey, Iraq, Armenia, and the Republic of Azerbaijan. In addition, it is also bordered by Iranian provinces such as East Azerbaijan, Zanjan and Kurdistan (Fig. 1). According to information obtained from Forests, Range and Watershed management organization, West Azerbaijan Province have five types of Micro-climates, including Highly semi-arid (HSA), Moderate semi-arid (MSA), Slight semi-arid (SSA), semi-wet (SW) and wet (W).

### Distribution of potential West Nile Vectors in West Azerbaijan Province

Mosquitoes were collected during May–Nov 2015 from 24 localities (wetlands) across the province (Table 1). Adults and larvae were collected from different habitats using standard methods (43). Collected samples were quickly identified using a stereo microscope and a standard morphological key at the collection sites (44).

**Table 1.** Collection sites of mosquitoes in West Azerbaijan Province, northwestern Iran, 2015

**County**	**Collection site**	**Longitude**	**Latitude**	**Altitude**	***Ae. caspius***	***An. maculipennis***	***Cx. pipiens***	***Cx. theileri***	***Cs. longiareolata***
**Bazargan**	Bazargan	44.38944	39.38833	1400	0	25	1	13	0
	Yarim-Ghiye	44.43656	39.44604	1409	175	65	0	420	0

**Khoy**	Hashiyeh rood	45.06315	38.57117	1058	3	0	6	18	0

**Mahabad**	Mahabad	45.71667	36.75	1351	0	10	89	25	4
	Khoor-khooreh	45.72341	36.98732	1279	205	2	120	0	30
	Kani barazan-wetland	45.77716	36.98689	1275	0	0	68	1	0
	Hajib khosh	45.79718	36.90788	1288	0	0	136	0	0
	Gapis	45.74795	36.93805	1286	0	0	4	7	0
	Beytas	45.69427	36.67645	1396	3	0	28	39	0

**Makoo**	Makoo	44.43333	39.3333	1411	6	7	82	23	3
	Sangar	44.43429	39.31578	1355	288	132	100	169	90
	Milan	44.4332	39.34351	1445	37	115	25	22	0
	Keshmesh tappeh	44.40093	39.33351	1385	0	0	150	94	0
	Glik gadim	44.66767	39.71264	807	31	0	26	0	0
	Deim-Gheshlagh	45.07119	39.34766	784	0	58	0	0	0
	Deimgeshlag	44.7987	39.62488	797	190	0	20	0	0

**Miandoab**	Miandoab	46.06947	36.98592	1291	0	0	98	0	0

**Naghadeh**	Naghadeh	45.41667	36.95	1338	0	56	0	0	7
	Yadegarloo	45.52839	37.03822	1284	0	10	0	23	19

**Plodasht**	Poldasht	45.07111	39.34778	788	0	10	55	89	0
	Khol-kholeh	44.74524	39.67784	784	0	3	0	40	0
	Ghooch-Ali	44.74391	39.66296	1401	0	25	1	5	0

**Sardasht**	Sardasht	45.48333	36.15	1556	0	18	0	0	0

**Urmia**	Urmia	45.05	37.66667	1328	0	128	151	163	246
	Naz-loo	44.98442	37.65172	1358	0	0	441	67	10
	Ghahraman-loo	45.17485	37.64896	1000	170	0	24	0	0
	Koor-Abad	44.64239	37.72749	1545	0	68	34	0	6
	Silvana	44.85142	37.42867	1577	0	120	0	0	0
	Gojar	44.83373	39.48785	1736	0	7	15	19	7
	Mavana	44.79643	37.56658	1617	0	13	0	32	24
	Shaharchay dam	44.98628	37.4952	1433	0	11	0	10	3
	Talebin	44.83398	37.54025	1608	0	0	0	149	0

### Climatic and environmental data

**Fig. 2.**
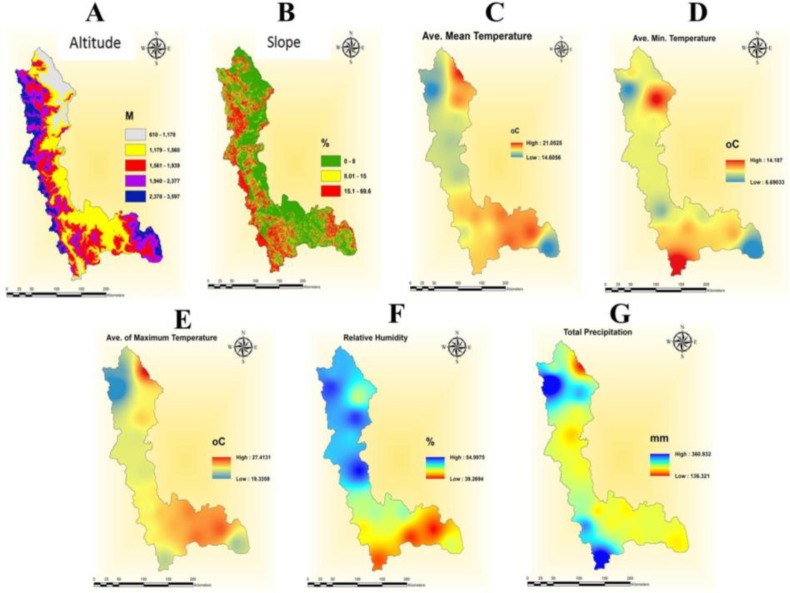
Environmental and meteorological variables affecting mosquito distribution in West Azerbaijan Province, northwestern Iran, (A): Altitude, (B): Slope, (C): Average of Mean Temperature, (D): Average of Minimum Temperature, (E): Average of Maximum Temperature, (F): Relative Humidity and (G): Total Precipitation.

As distribution of mosquitoes and their transmitted diseases are notably dependent on environmental and climatic factors, the effective climatic data, including maximum monthly temperature, minimum monthly temperature, mean monthly temperature, mean relative humidity and rainfall data of decade (2004–2014) were obtained from 16 stations of West Azerbaijan Meteorological Organization (Table 2). Datasets at district level were created in Excel sheets for further analysis by ArcGIS 10.3. The pattern of recent decade of Maximum, Mean and Minimum temperature of different areas in West Azerbaijan Province was analyzed and mapped in Fig. 2 and summarized in Table 2.

**Table 2.** Average of environmental variables in meteorological stations of West Azerbaijan Province of Iran, 1997–2016

**Localities**	**Geographical properties**				**Environmental variable**								

	**Longitude**	**Latitude**	**Altitude**	**Total Rainfall**	**Mean Relative Humidity**	**Mean Temp**	**Min Temp**	**Max Temp**
**Bookan**	46.21667	36.53333	1386.1	360.5	46	14.5	6.3	19.9
**Chaldoran**	44.38333	39.06667	1788	449.5	58	9.2	2.7	13.9
**Ghareh Ziahadin**	45.01667	38.9	1108	357.3	52	13.6	8	18
**Khoy**	44.96667	38.55	1103	289.2	59	12.1	5.5	18.6
**Mahabad**	45.71667	36.75	1351.8	403.8	53	13.0	6.9	19.15
**Makoo**	44.43333	39.3333	1411.3	302.8	57	10.6	5.5	15.6
**Miandoab**	46.05	36.96667	1300	303.8	53	14.4	5.6	20.0
**Naghadeh**	45.41667	36.95	1338	339.0	53	13.9	5.8	19.2
**Oshnavie**	45.13333	37.05	1415.9	437.3	52	13.5	4.5	18.6
**Piranshahr**	45.15	36.7	1443.5	672.7	52	12.0	6.2	17.9
**Poldasht**	45.07119	39.34778	787	198.8	51	14.7	4.8	20.2
**Salmas**	44.85	38.21667	1337	247.5	57	11.5	5.2	17.8
**Sardasht**	45.48333	36.15	1556.8	841.2	49	13.1	9.2	16.8
**Shahindej**	46.73333	36.6667	1395	334.8	47	14.9	6.7	20.4
**Takab**	47.1	36.4	1817.2	338.6	55	9.4	2.5	16.4
**Urmia**	45.05	37.66667	1328	338.9	61	11.6	5.4	17.7

### Spatial Analysis

For proper analysis, the data regarding distribution of mosquitoes across West Azerbaijan Province were acquired from previous studies (30, 33) and added to the findings of this survey in database created in ArcMap. Inverse distance weighted (IDW) interpolation analysis was conducted on the data of synoptic stations to find the climatic variables in the collection sites of different mosquito species. This analysis interpolates a raster surface from points and estimates cell values by averaging the values of nearby sample data points. The closer a point is to the center of the cell which is being estimated, the more weight it is given. The equation for IDW analysis is:

$$\hat{\nu }=\frac{\sum_{i=1}^{n}\frac{1}{d_{i}}\nu_{i}}{\sum_{i=1}^{n}\frac{1}{d_{i}}}$$

where

*v̂*= value to be estimated
*v*_i_= known value
d_i_..., d_n_= distances from the n data points to the point estimated n

**Fig. 3.**
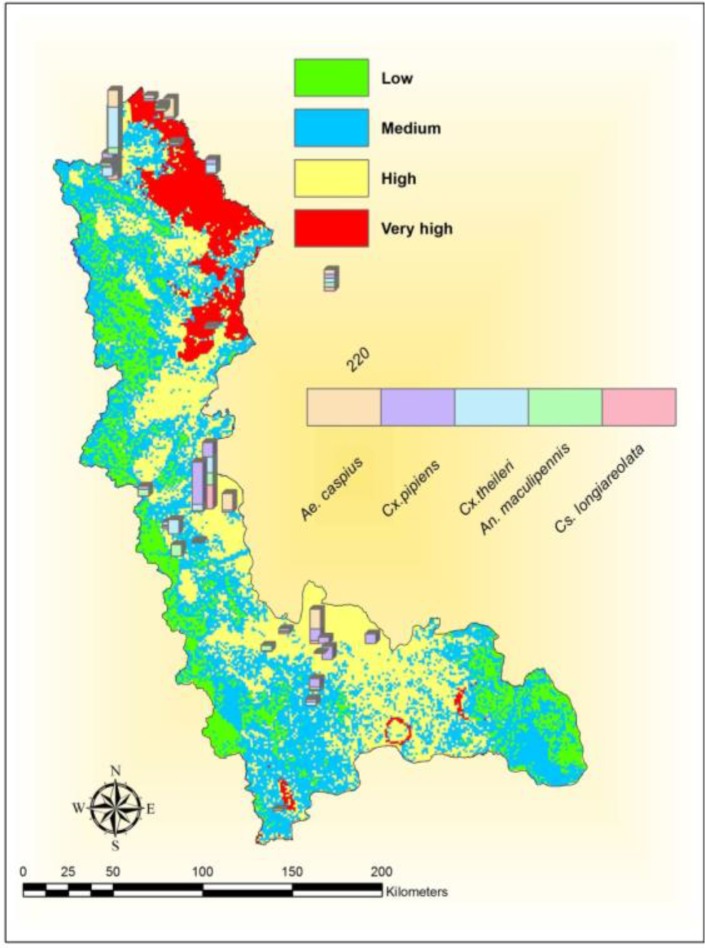
Spatial distribution of mosquitoes and hot spots for vectors of West Nile virus in West Azerbaijan Province regarding environmental factors affecting the transmission of WNV, northwestern Iran, 2015

Layers of environmental factors that are important in transmitting the WNV were categorized and weighted based on their effects on disease transmission, and important impact on vector-borne diseases (3, 45–47) (Table 3). The categorized and weighted environmental factors were overlaid with the vectors distribution across the West Azerbaijan Province for determination of high-risk areas for the establishment of the transmission cycle of WNV based on the mentioned environmental factors. As the five species (*Ae. caspius*, *An. maculipennis*, *Cs. longiareolata*, *Cx. pipiens* and *Cx. theileri*) reported from study area, also are known proven and suspected vectors of WNV in different parts of the world, they have been included in final analysis (Fig. 3).

**Table 3.** Categorizing the environmental factors based on their effects on disease transmission, and important impact on vector-borne diseases (3, 45–47)

**Environmental Factor**	**Ratio**	**Standard weight**	**Category**	**Group**	**Effect on diseases Transmission**
**Mean Temperature (°Celsius)**	55	0.55	26–30	4	Very High
			20–26	3	High
			18–20	2	Medium
			14–18	1	Low

**Relative Humidity (%)**	35	0.35	60<	4	Very High
			50–60	3	High
			40–50	2	Medium
			<40	1	Low

**Altitude (m)**	5	0.05	0–800	4	Very High
			800–1200	3	High
			1200–2000	2	Medium
			<2000	1	Low

**Slope (%)**	5	0.05	<8	3	Very High
			8–15	2	Medium
			15<	1	Low

### Ethics approval and consent to participate

Prior to the approval of all projects by the Urmia University of Medical Sciences (UMSU), they are reviewed and endorsed by the Ethics Committee of the UMSU. Sample collection was carried out from private human and animal dwellings. At least one day prior to any sample collection, the owners were informed by the Local Health System officers. The whole process was coordinated, managed and documented by the “Local Health System officer” in the study areas.

## Results

Overall, 2813 mosquito specimens from different regions of the province were collected and identified (Table 1). Analysis of some climatic variables across the collection sites for different mosquito species is summarized in Table 4. The most frequent species in our study was *Cx. pipiens* (22 out of 24 collection sites), while *Ae. caspius* was found only in 10 localities.

**Table 4.** Average of some environmental and climatic variables in the collection sites of mosquitoes, West Azerbaijan Province, northwestern Iran, 2015

**Species**	**No. of collection sites**	**Precipitation (mm)**	**Average of Altitude (M)**	**Average of Relative Humidity (%)**	**Average of Maximum Temperature (°C)**	**Average of Minimum Temperature (°C)**	**Average of Mean Temperature (°C)**
***Ae. caspius***	10	326.3	1214.7	56	17.21	5.61	11.74
***An. maculipennis***	20	347.3	1273.9	54.91	17.75	5.66	12.4
***Cx. pipiens***	22	328.6	1227.5	55.33	17.89	5.6	12.3
***Cx. theileri***	21	330.65	1275.1	55.78	17.65	5.6	12.1
***Cs. longiareolata***	12	345.1	1364.25	56.14	17.95	5.66	12.3

The altitudinal activity of suspected vectors was determined across the areas with an average of 1214m for *Ae. caspius* to 1364m for *Cs. longiareolata* (Table 4). Considering the altitude of the study area which ranges from 605m to 3600m, the values of the altitude were classified into five categories, by overlying the presence of potential vectors and their distribution map on altitude values of the province, height less than 1615m (605–1500m), are more prepared and favored places for mosquitoes in the study areas. Finally using the scale of the most important environmental variables (Table 3), determination of high risk areas for WNV and distribution of potential vectors across the studied areas was analyzed and revealed in Fig. 3. The map of the mosquitoes including proven and suspected vectors of WNV, ie *Cx. pipiens*, *Cx. theileri* and *Ae. caspius* were collected mostly in hot spots, determined by the model (Fig. 3).

## Discussion

The study area has a large number of wet-lands that host of migratory birds. Although WNV is an enzootic disease among birds (48), however, humans transmission is possible through bites of infected mosquitoes (49). WNV is responsible for disease outbreaks among human in the United States, Europe, and the Middle East (50). It has been detected in recent studies from birds, horse, human and mosquitoes in Iran (31–36).

In the present study, environmental factors affecting the transmission of WNV were studied and high-risk areas were determined (Fig. 3). Earlier study on the impact of climate and environmental variables on WNV in Iran, using data from seropositive horses, found four studied factors that correlated with WNV infection in equine, these factors were temperature, distance to wetlands, and local and regional Normalized Difference Vegetation Index (NDVI) (27). Results from previous studies also indicated the presence of WNV vectors (*Cx. pipiens*, *Cx. theileri*, *Ae. caspius*) in the region (36, 40) and their infection with WNV in Iran (36). In addition, the studied areas have the potential for establishment of WNV transmission. The results of current study regarding the determination of the high-risk areas are interesting because they are in accordance with results from previous study that isolated West Nile virus from vectors (36). The isolation of the virus in the mentioned study has been reported from the high-risk areas identified in current study and this point shows the acceptable accuracy of the results of the current study which analyzed environmental factors (temperature, relative humidity, Altitude and Slope). These areas should be considered in planning of WNV epidemic control programs. Among the probable reasons increasing the risk of establishing transmission cycle of WNV in these areas is the wide range of wetlands in the area and the presence of migratory birds as the reservoirs of disease and also the appropriateness of environmental conditions for the presence and abundance of various species of mosquitoes, as potential vectors of WNV. Also it seems northeastern areas of the province, which have lower altitudes and slopes with warmer temperatures and more water bodies, were found to have better condition for the activity of mosquitoes.

Diversity of environmental conditions in West Azerbaijan Province, which provides suitable environment for the establishment of various species of mosquitoes (Table 1) have been found in this current and related studies (36, 40, 42). Other results have shown the effect of temperature in transmission of WNV by *Cx. pipiens*, and the effect of temperature on the replication of the virus within the mosquito’s body and the incubation period, as well (51). WNV has the ability to replicate in mosquitoes in wider range of temperatures between 14 °C in mosquitoes (52) to 45 °C in birds (53). By increasing the temperature, WNV propagation rate could be increased.

The suitable temperature for WNV replication is provided in the northeastern and southern parts of the provinces of Iran. Therefore, these areas need more investigation on blood feeding pattern of mosquitoes and their infection with WNV. Recent study on the feeding patterns of potential WNV vectors in South-West Spain showed that *Cx. modestus*, *Cx. perexiguus* and *Cx. pipiens* mainly feed on birds, while *Cx. theileri* and *Ae. caspius* mainly feed on mammals. *Cx. perexiguus* had the highest potential for enzootic virus transmission, followed by *Cx. modestus* and *Cx. pipiens*. According to results of the South-West Spanish study, potential transmission risk to humans was low for *Cx. pipiens*, *Cx. theileri* and *Ae. caspius* (54). The frequency of feeding on humans was only affected by season, while the low number of human blood meals was related to their study site. On the other hand, the researchers worked in natural areas with very low anthropic presence (54). Most of these mosquito species have been reported from our study area as well (36), therefore, they may have some roles in both avian to avian enzootic cycle and avian-to-mammal transmission.

Certainly, the current study did not cover all important environmental factors affecting the potential of WNV transmission regarding the environmental factors and the effect of other important factors such as wetland, Normalized Difference Vegetation Index (NDVI) and land use should be analyzed and determined in future studies.

## Conclusion

The present study serves as a preliminary guide that shows the important effects of environmental conditions on one of the important members (mosquitoes) in the transmission cycle of WNV and should be continued with supplementary studies. Taking into account the vector bio-ecologic conditions, other environmental factors and the interaction of the virus and the vectors as well as other important rings in the transmission of disease, the data obtained from this current study will be very useful and effective in knowing the exact nature of the disease transmission pathways and help in designing its control strategies.
